# An image dataset for use in detecting unwanted bolt rotation

**DOI:** 10.1016/j.dib.2025.111788

**Published:** 2025-06-16

**Authors:** Tom Bolton, Julian Bass, Tarek Gaber, Taha Mansouri

**Affiliations:** University of Salford, Department of Science, Engineering, and Environment, The Crescent, Salford, M5 4WT, United Kingdom

**Keywords:** Continuous maintenance, Bolt rotation, Change detection

## Abstract

In any industrial system, ensuring that the engineered components therein are in working order is essential for the safety of workers and for efficient and cost-effective running. However, due to factors such as stress, deformation, and corrosion, individual components degrade over time, eventually leading to failure.

Whilst there exist several public training datasets for use in bolt detection, there is none in the area of bolts or other mechanical fixings changing over time. We prepared a novel dataset of over 1,100 images depicting a bolted apparatus from different angles, and with varying degrees of bolt rotation. The images were taken in laboratory conditions, with carefully measured variations. As far as we know, no other such dataset exists.

Specifications Table

**Table:** 

Subject	*Computer Vision and Pattern Recognition*
Specific subject area	Computer vision for continuous maintenance in industrial applications. Data acquisition, deep learning, metric loss.
Type of data	Image, JPEG.
Data collection	We created an apparatus having five M20 bolts and photographed it with three of the bolts progressively rotated counterclockwise. The images were taken using a tripod mounted Pentax K-7 DSLR camera and were collected in controlled conditions in a mechanical engineering laboratory at the University of Salford. 1,112 images were collected depicting the apparatus at various camera angles and, using a zoom lens, four different focal lengths.
Data source location	University of Salford, Department of Science, Engineering, and Environment, The Crescent, Salford, M5 4WT, United Kingdom
Data accessibility	Repository name: A dataset depicting simulated bolt rotation Data identification number: 10.6084/m9.figshare.25393780 Direct URL to data: figshare.com/ndownloader/files/44996119 Instructions for accessing these data: none
Related research article	

## 1 Value of the Data

• The bolt rotation dataset offers a set of images depicting gradual loosening of mechanical connections from which temporal pairs or triplets might be constructed. Researchers may find this data interesting as it can offer a simulation of a condition that might otherwise take many weeks or months to observe naturally.
• The bolt rotation dataset is publicly available, and annotated with degrees of deviation in bolt rotation, camera angle, focal length, and camera height. One of the goals in publicly releasing this data was to enable researchers to move from detection of static objects, to change over time using comparative deep learning methods.
• The dataset was constructed to include varying degrees of geometric noise – camera angle and focal length – as well as bolt rotation.
• As far as we are aware, no other such dataset is available that depicts bolt rotation. We believe the data has applications in machine learning for continuous maintenance and anomaly detection, as well as remote observation using robots or drones.

## 2 Background

Bolts and bolting are commonly used as a method of securing parts of structures to one another. To ensure integrity of a connection, the bolts must be secured with a sufficient preload force to provide a rated level of mechanical security. Research has moved towards the use of machine learning to identify faulty bolted connections [1,2].

For static object recognition tasks, very few annotated bolt image datasets are available. NPU-Bolt comprises 337 images, annotated with 1275 individual bounding boxes [3] and has been used to research deep learning models for bolt recognition [4]. For detecting change in bolt rotation angle over time, we provide a dataset of images depicting a bolted apparatus with a clear sequence of rotation of several of the bolts. At each degree of rotation, the apparatus was photographed at different camera angles, different heights, and four different focal lengths resulting in a set of 1,112 images. The ability to construct temporal pairs or triplets of images with this dataset for use in metric loss deep learning networks, simulating unwanted bolt rotation over time, is what makes this collection unique.

## 3 Data Description

We curated the bolt rotation dataset in 2023. We constructed an apparatus that simulates a bolted connection – this comprised some sheet material to which five bolts were affixed, each of which could be rotated to represent unwanted loosening. We captured 1,112 images divided into 12 classes, each representing a five-degree deviation of rotation in the lower three bolts from a known starting point.

**Fig. 1:**
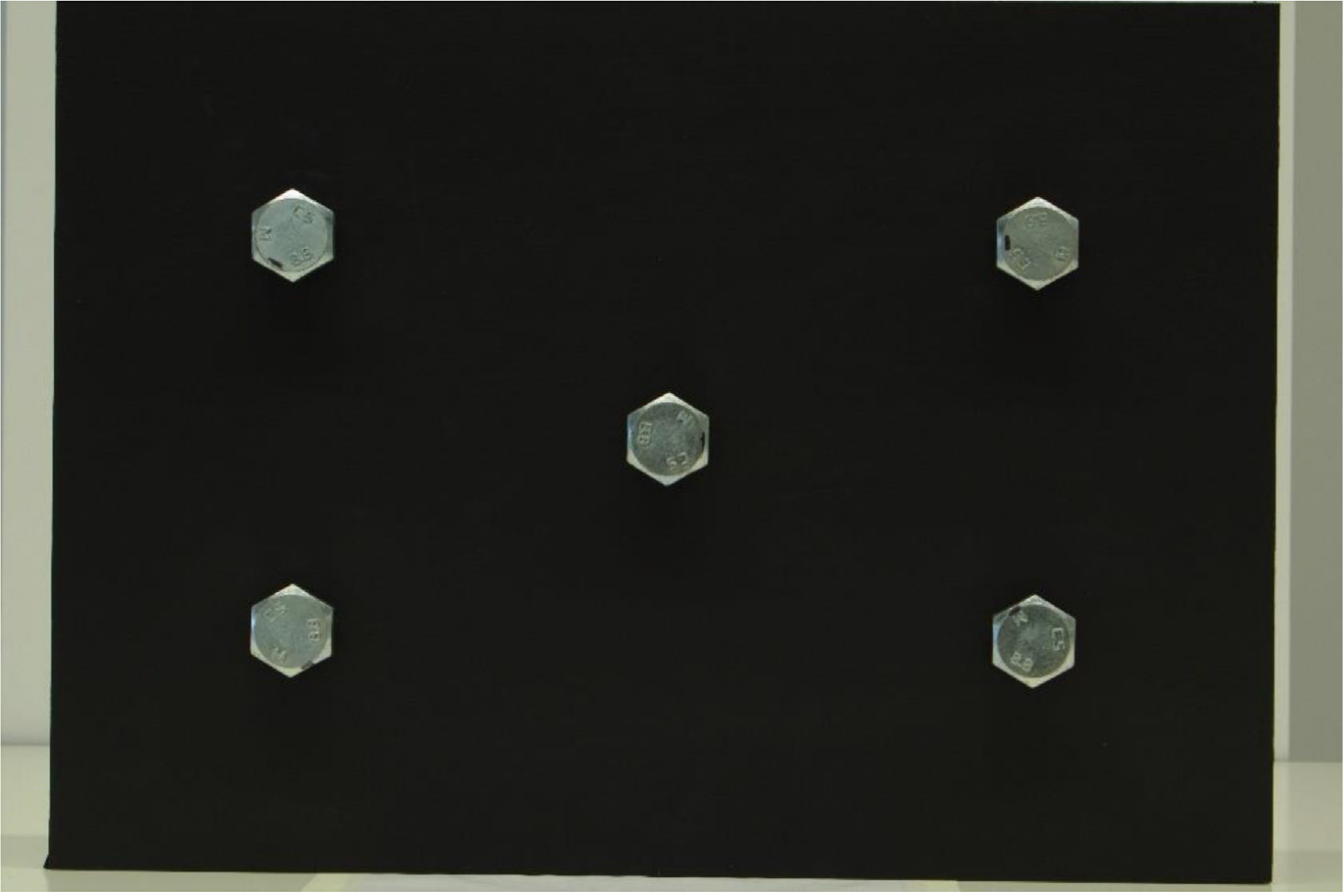
bolted apparatus showing bolts in initial conditions – 0° bolt rotation, 0° camera angle deviation, 1040mm camera height, 80mm focal length.

With each of the five bolts initially positioned such that two of the six sides of the head were vertical, as shown in Fig. 1, the lower three bolts were progressively rotated counterclockwise in five-degree increments.

Table 1 details the hierarchy and structure of the captured images. The apparatus was photographed from two heights: 1040mm (level with the apparatus) and 1590mm (looking down upon the apparatus).

**Table 1:** structure and hierarchy of the dataset.

Camera Height (mm)	Bolt Rotation (Degrees)	Camera Angle Deviation (Degrees)	Focal Length (mm)
1040	0	0, 5, 10, 15, 20, 25, 30, 35, 40, 45, 50, 55, 60, 65, 70, 75, 80, 85, 90	80, 50, 35, 28
	5	0, 5, 10, 15, 20, 25, 30, 35, 40, 45, 50, 55, 60, 65, 70, 75, 80, 85, 90	80, 50, 35, 28
	10	0, 5, 10, 15, 20, 25, 30, 35, 40, 45, 50, 55, 60, 65, 70, 75, 80, 85, 90	80, 50, 35, 28
	15	0, 5, 10, 15, 20, 25, 30, 35, 40, 45, 50, 55, 60, 65, 70, 75, 80, 85, 90	80, 50, 35, 28
	20	0, 5, 10, 15, 20, 25, 30, 35, 40, 45, 50, 55, 60, 65, 70, 75, 80, 85, 90	80, 50, 35, 28
	25	0, 5, 10, 15, 20, 25, 30, 35, 40, 45, 50, 55, 60, 65, 70, 75, 80, 85, 90	80, 50, 35, 28
	30	0, 5, 10, 15, 20, 25, 30, 35, 40, 45, 50, 55, 60, 65, 70, 75, 80, 85, 90	80, 50, 35, 28
	35	0, 5, 10, 15, 20, 25, 30, 35, 40, 45, 50, 55, 60, 65, 70, 75, 80, 85, 90	80, 50, 35, 28
	40	0, 5, 10, 15, 20, 25, 30, 35, 40, 45, 50, 55, 60, 65, 70, 75, 80, 85, 90	80, 50, 35, 28
	45	0, 5, 10, 15, 20, 25, 30, 35, 40, 45, 50, 55, 60, 65, 70, 75, 80, 85, 90	80, 50, 35, 28
	50	0, 5, 10, 15, 20, 25, 30, 35, 40, 45, 50, 55, 60, 65, 70, 75, 80, 85, 90	80, 50, 35, 28
	55	0, 5, 10, 15, 20, 25, 30, 35, 40, 45, 50, 55, 60, 65, 70, 75, 80, 85, 90	80, 50, 35, 28

1590	0	0, 10, 20, 30, 40, 50, 60, 70, 80, 90	80, 50, 35, 28
	10	0, 10, 20, 30, 40, 50, 60, 70, 80, 90	80, 50, 35, 28
	20	0, 10, 20, 30, 40, 50, 60, 70, 80, 90	80, 50, 35, 28
	30	0, 10, 20, 30, 40, 50, 60, 70, 80, 90	80, 50, 35, 28
	40	0, 10, 20, 30, 40, 50, 60, 70, 80, 90	80, 50, 35, 28
	50	0, 10, 20, 30, 40, 50, 60, 70, 80, 90	80, 50, 35, 28

The apparatus was then photographed with the lower three bolts progressively rotated counterclockwise. At each of these points the bolted apparatus is depicted at 19 different camera angles, each denoting a five-degree rotation from 0° (perpendicular to the camera) to 90° at which point the apparatus is side-on to the camera. The result – 0 to 55° in five-degree increments – gives us 12 primary classes. Each image belongs to one class. Each class has either four or eight images.

Every time the apparatus was photographed at a given height, bolt, or camera angle, four different focal lengths (80mm, 50mm, 35mm, 28mm) were used to simulate the subject’s varying distance from the camera.

**Fig. 2:**
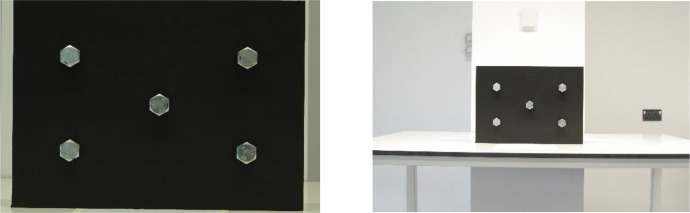
a and b: change in focal length from 80mm (a) to 28mm (b) – camera angle deviation, camera height, bolt rotation unchanged.

**Fig. 3:**
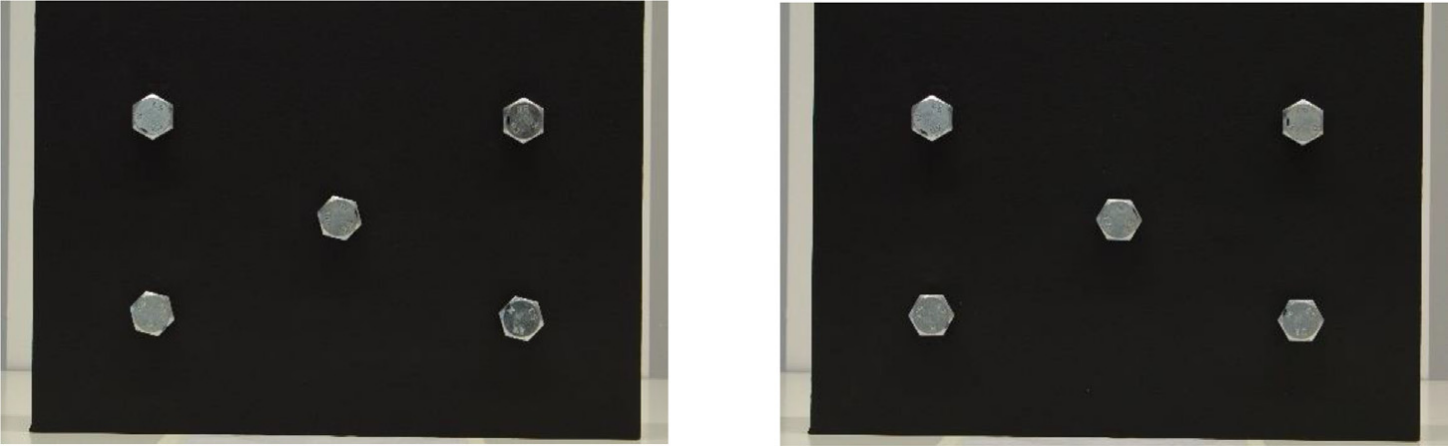
a and b: change in bolt rotation deviation from 15° (a) to 30° (b) – camera angle deviation, camera height, focal length unchanged.

**Fig. 4:**
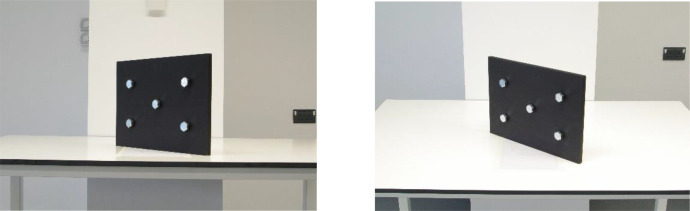
a and b: change in camera angle (horizontal) from 30° (a) to 40° (b) and camera height (vertical) from 1040mm (a) to 1590mm (b) – bolt rotation and focal length unchanged.

Sample images can be seen in Figs. 2a and b, 3a and b, and 4a and b. Depicted are changes in focal length (Fig. 2a and b), changes in bolt rotation (Fig. 3a and b) and changes in both camera angle and height (Fig. 4a and b).

**Fig. 5:**
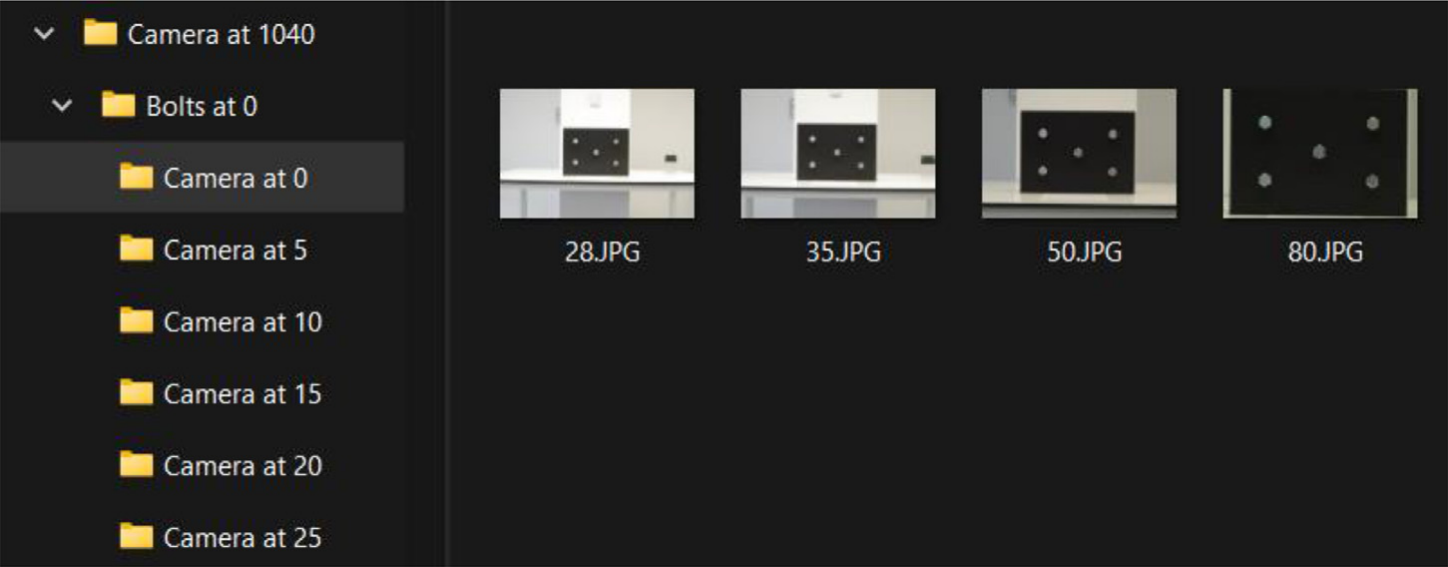
the structure of the dataset on disc as shown in Windows File Explorer.

Fig. 5 shows a partial view of the dataset as represented on the storage medium. The files are organized to follow the structure and hierarchy outlined in Table 1 with camera height, and deviations in bolt rotation angle and camera angle encoded into the folder names. The image filenames represent the focal length of the camera lens. Included with the dataset is a JSON file that carries parameter information for each file in the collection.

## 4 Experimental Design, Materials and Methods

An apparatus was constructed from 18mm plywood, painted matt black and measuring 450mm in width and 325mm in height. Threaded into the plywood were five M20 bolts – metric (“M”) thread, 20mm in diameter. The bolts were threaded into holes in the apparatus – one was placed at each corner, 90mm from each edge. A fifth bolt was placed in the centre of the apparatus.

In addition to providing a useful baseline for research, this data was collated for training metric loss machine learning architectures designed by the authors, who hypothesised that smaller rotations would be more difficult to detect. How small the rotation deviation would need to become to cause the machine learning to struggle was unknown beforehand. This was the main driver behind choosing a spread of rotation amounts that covered 60°, beyond which images of a six-sided bolt head would repeat. For testing with metric loss, the authors wanted images that were consistent in parameters other than bolt rotation, camera angle, and focal length. Differences in lighting were avoided to ensure that as little noise as possible was learned by the algorithms, and for the same reason identical bolt types were used throughout.

The images were captured over several days in a general-use mechanical engineering laboratory at the University of Salford; the background is plain in colour and the lighting was artificially controlled. The camera used was a Pentax K-7 DSLR with a 28-80mm zoom lens. The images were captured at a resolution of 4672 × 3104 pixels in both RAW and JPEG format. Due to space considerations, only the JPEG images are included in the public dataset.

**Fig. 6:**
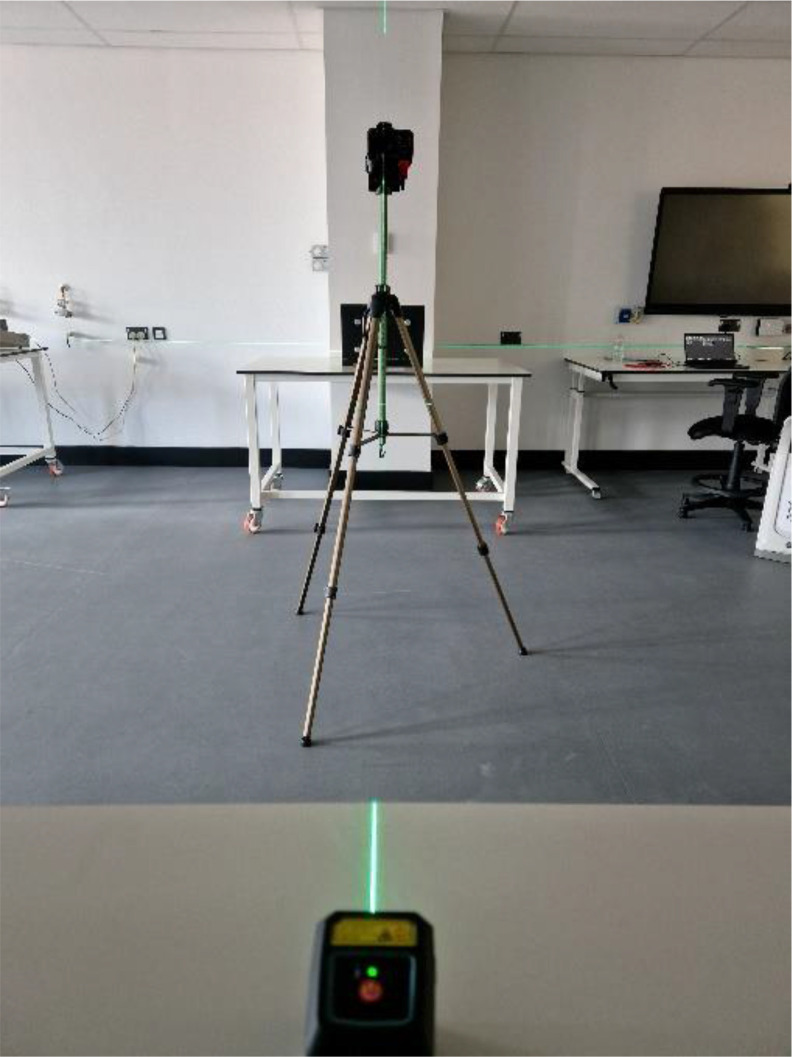
horizontal aligning of the camera with the apparatus using a laser.

The laboratory setup can be seen in Fig. 6. The tripod was set true to the apparatus using square and a laser.

**Fig. 7:**
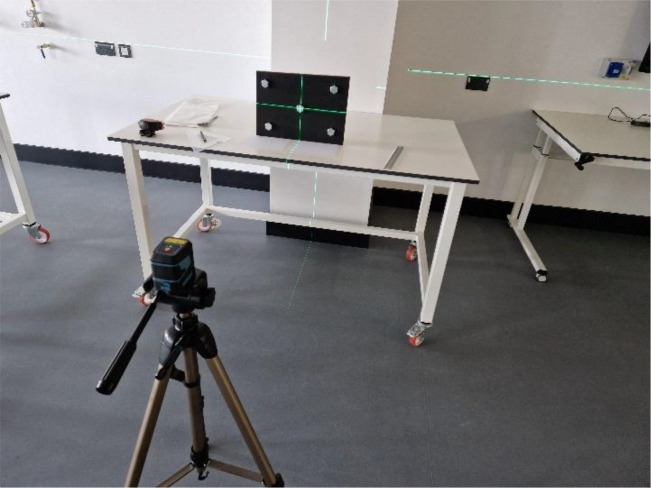
focusing the camera on the centre of the apparatus – horizontally and vertically – using a laser.

As can be seen in Fig. 7, the camera was focused on the central bolt – again, checked with a laser. Images were captured with the camera’s lens at the same height as the central bolt (1040mm from floor level) and with the camera raised above – and looking down upon – the central bolt from a height of 1590mm from floor level. The camera was sited 1,610mm from the apparatus.

The laboratory lighting was provided by overhead strip lights. These were carefully matched during several days of capturing images to ensure that the levels were as consistent as possible.

## Limitations

Our measurements in these experiments were accurate to one degree. With a more precisely machined apparatus, for example using a metal substrate machined with computer numerically controlled (CNC) equipment, we may have captured bolt rotation of one degree instead of using increments of five degrees.

We did not consider other factors that may lead to failure of a bolted connection such as corrosion or stress. The authors’ machine learning research was centred around bolt rotation in isolation to address a specific industrial problem. Further work can, and should, address these limitations.

## Ethics Statement

The authors have read and followed the requirements for publishing in Data in Brief. No person, animal, or social media data was collected. These experiments form part of a PhD project for which ethical clearance was formally obtained from the University of Salford.

## CRediT authorship contribution statement

**Tom Bolton:** Conceptualization, Methodology, Data curation, Validation, Resources, Writing – original draft, Visualization. **Julian Bass:** Conceptualization, Writing – review & editing, Supervision, Project administration, Funding acquisition. **Tarek Gaber:** Conceptualization, Writing – review & editing, Supervision, Project administration. **Taha Mansouri:** Conceptualization, Writing – review & editing, Project administration.

## Data Availability

FigshareA dataset depicting simulated bolt rotation (Original data)
